# Clinical Characteristics of Hospitalized Patients with Drug-Induced Acute Kidney Injury and Associated Risk Factors: A Case-Control Study

**DOI:** 10.1155/2020/9742754

**Published:** 2020-09-14

**Authors:** Chengxuan Yu, Daihong Guo, Chong Yao, Hongyi Yang, Siyuan Liu, Yu Zhu, Xianghao Kong

**Affiliations:** ^1^Pharmacy Department, Medical Security Center, Chinese PLA General Hospital, Beijing 100853, China; ^2^Graduate School, Chinese PLA General Hospital, Beijing 100853, China; ^3^College of Pharmacy, Chongqing Medical University, Chongqing 40016, China

## Abstract

**Background:**

Drug-induced acute kidney injury (D-AKI) is increasingly common and can extend the hospital length of stay and increase mortality. This study is aimed at analyzing the clinical characteristics of hospitalized patients with D-AKI and the associated risk factors in a multidrug environment.

**Methods:**

A retrospective study among hospitalized patients was conducted in July 2019 based on the Adverse Drug Events Active Surveillance and Assessment System-2 developed by the authors. Four controls were matched with each case according to the matching criteria. The risk factors for D-AKI were identified by binary multivariate logistic regression.

**Results:**

A total of 23,073 patients were hospitalized in July 2019, 21,131 of whom satisfied the inclusion criteria. The independent risk factors for D-AKI consisted of alcohol abuse (odds ratio (OR), 2.05; 95% confidence interval (CI), 1.04-4.07), nonsteroidal anti-inflammatory drug (NSAID) use (OR, 2.39; 95% CI, 1.25-4.58), diuretic use (OR, 2.64; 95% CI, 1.42-4.92), prior anemia (OR, 4.10; 95% CI, 1.94-8.67), and prior chronic kidney disease (OR, 2.33; 95% CI, 1.07-5.08).

**Conclusions:**

The occurrence of D-AKI in hospitalized patients had significant associations with alcohol abuse, combination therapy with NSAIDs or diuretics, and prior anemia or chronic kidney disease. Clinicians should meticulously follow patients with the above characteristics.

## 1. Introduction

Drug-induced acute kidney injury (D-AKI) refers to kidney injury caused by drugs or their metabolites within 7 days after the use of one or more drugs [1]. D-AKI is increasingly recognized as a relatively common adverse drug reaction (ADR) in clinical practice. It can be caused by various medications through complicated pathogenic mechanisms and has been associated with high morbidity and mortality [2–4]. D-AKI accounts for 18% to 27% of AKI cases in US hospitals [5, 6]. In China, the percentage of D-AKI among all AKI cases has increased from 26.5% to 42.9% [7, 8]. A Chinese multicenter AKI survey showed that 71.6% of patients with AKI had a history of potentially nephrotoxic drug use before or during renal injury [9].

Drugs are not the only risk factor for AKI; other possible risk factors for D-AKI include age (>60 years old), hypertension, chronic kidney disease (CKD), and diuretic therapy [10, 11]. Identifying patients at risk for D-AKI can help make effective use of medical resources during hospitalization to develop prevention strategies for D-AKI and promote patient safety. However, the assessment of risk factors in recent studies was mostly based on the multivariate analysis of specific patients [2, 12, 13]. Sample collection was limited by disease and age, and drug use was mostly nontherapeutic, so the distribution of risk factors among all the inpatients using drugs could not be obtained, especially in the Chinese population.

Therefore, based on the Adverse Drug Events Active Surveillance and Assessment System-2 (ADE-ASAS-2) developed by the Chinese People's Liberation Army (PLA) General Hospital, we carried out a case-control study to explore the clinical characteristics of patients with D-AKI and its risk factors to assess the prevention of D-AKI.

## 2. Materials and Methods

### 2.1. Study Design and Patient Selection

We conducted a case-control study in a group of hospitalized patients over the age of 18 to explore the features of D-AKI. Eligible patients included those who developed AKI after treatment with nephrotoxic drugs in the Chinese PLA General Hospital in July 2019.

Data of all patients were obtained from the hospital information system (HIS), including demographic, admission diagnosis, prescription, previous medical history, and laboratory examination data. The ADE-ASAS is based on trigger technology and text recognition technology, which can be connected to the HIS to extract patient information. Intelligent active surveillance of a single drug in multiple ADR modules can be accomplished [14, 15]. The accuracy of this system has been evaluated in multiple studies, with a positive predictive value reaching 69.4% [15, 16]. The ADE-ASAS-2 is based on the same principle with different algorithms to monitor all inpatients with all medication conditions. The main operating process of the ADE-ASAS-2 system was as follows: first, a monitoring plan was established, including the basic information (name of the plan, the monitoring time and the monitoring age range, etc.), the monitored drug (not set), the monitoring module (D-AKI module), and the monitoring ward (not set). Second, the D-AKI module was entered to set the monitoring criteria, including the exclusion criteria, inclusion criteria, and definition of severity (not set). Finally, the monitoring plan was initiated. Once the monitoring indicators triggered the inclusion criteria, the system could preliminarily determine whether a patient had developed AKI and issue early warning signals. Because no monitored drugs were set up, drugs that are time-related to the increase in serum creatinine (SCr) could be captured as D-AKI early warning signals. An alarm case may include multiple early warning signals. Then, two clinical pharmacists evaluated the alarm results consecutively, and cases with inconsistent assessment results were referred to an expert for final judgment to determine whether D-AKI had occurred. If patients had multiple positive signals during hospitalization, only the first signal was assessed.

According to the AKI SCr definition criteria of 2012 Kidney Disease: Improving Global Outcomes (KDIGO) Clinical Practice Guidelines for Acute Kidney Injury [17], the inclusion criteria were as follows: (1) age ≥ 18 years old, (2) a full prescription model (temporary prescriptions and long-term prescriptions), and (3) an increase in SCr by at least 0.3 mg/dL within 48 hours or an increase in SCr to at least 1.5 times higher than baseline within the prior 7 days. The exclusion criteria were as follows: (1) an absent baseline SCr measurement or (2) baseline SCr > 5 mg/dL. Two clinical pharmacists independently evaluated the cases who elicited warning signals. The other exclusion criteria were as follows: (1) patients with stage 5 CKD (*n* = 26), (2) patients with missing laboratory indexes within 7 days after medication administration (*n* = 48), (3) patients with incomplete clinical records (*n* = 65), or (4) patients undergoing dialysis or who underwent nephrectomy or kidney transplantation (*n* = 13). The Naranjo Adverse Drug Reaction Probability Scale (Naranjo Scale) was used to determine whether AKI could be caused by drugs [18]. The ADR was assigned to a probability category from the total score as follows: definite ≥ 9, probable 5 to 8, possible 1 to 4, and doubtful ≤ 0. Patients with scores ≥ 1 were defined as D-AKI. The definition in the KDIGO guidelines does not include an explanation of the etiology of AKI and lacks relevant clinical information. Thus, the classification of AKI is unclear.

Controls were randomly selected among patients hospitalized in July 2019, and four controls were matched to each case. The control matching criteria included the following: (1) no AKI, (2) use of the same nephrotoxic drug, (3) the same dose and administration routes, (4) a difference within 2 days of the total number of days of nephrotoxic drug exposure, and (5) a difference within 5 days of the length of stay. This study was approved by the Ethics Committee of Chinese PLA General Hospital. All patient data were kept strictly confidential.

### 2.2. Data Collection and Definitions

All the information of patients admitted to the Chinese PLA General Hospital in July 2019 was monitored and extracted from the HIS using the ADE-ASAS-2, including demographic data (age, gender, height, weight, body mass index (BMI), smoking history, alcohol abuse, hospital stay, and the number of concomitant drugs used); comorbidities (diabetes, hypertension, cardiovascular disease, anemia, and CKD); medication (angiotensin-converting enzyme inhibitors (ACEIs), angiotensin receptor blockers (ARBs), nonsteroidal anti-inflammatory drugs (NSAIDs), diuretics, and vancomycin); and laboratory test results (SCr, fasting blood glucose, uric acid (UA), hemoglobin (HB), red blood cell (RBC) count, white blood cell (WBC) count, platelet (PLT) count, and red blood cell distribution width (RDW)). Secondary data, such as the estimated glomerular filtration rate (eGFR), were calculated as needed. The number of concomitant drugs was calculated as the number of other medications during the period of use of suspected drugs during admission. Diabetes mellitus was defined as having at least 2 fasting blood glucose measurements > 7 mmol/L or the use of antidiabetic agents. Hypertension was defined as a previous diagnosis of hypertension, previous use of antihypertensive medications, or a systolic pressure > 140 mmHg and/or a diastolic pressure > 90 mmHg on at least 2 separate measurements during hospitalization [19, 20]. Cardiovascular disease included the diagnosis of congestive heart failure, myocardial infarction, and unstable angina pectoris. Anemia was defined as a baseline hemoglobin value below 130 g/L for men and 120 g/L for women [21]. Preexisting chronic kidney disease was defined as having an eGFR < 60 mL/min/1.73 m^2^ for 3 months with or without kidney damage or CKD explicitly mentioned in the admission diagnosis [22], and eGFR was calculated using the Chronic Kidney Disease Epidemiology Collaboration creatinine equation because it is more precise than the Modification of Diet in Renal Disease formula according to the recommendations of clinical practice guidelines [17, 23]. We defined the SCr baseline as the last laboratory measurement between 7 days before and 2 hours after administration of the suspected drug. The collection time of other laboratory values was the most recent laboratory measurement before the suspected drug was administered.

### 2.3. Statistical Analysis

SPSS statistical software (version 25.0; SPSS, IBM Corporation, USA) was used to statistically analyze the data obtained in the study. For the baseline characteristics, a one-way Kolmogorov-Smirnov test was used to determine the distribution patterns of continuous variables. Quantitative data with a normal distribution are represented as the means and standard deviations, and data with a nonnormal distribution are represented as medians and interquartile ranges. Qualitative data are displayed as numbers and percentages. The unpaired Student *t* test was used to compare normally distributed continuous variables, while the Mann–Whitney *U* test was used for group analyses when the distribution was not normal. The *χ*^2^ test or Fisher's exact test was used to compare categorical variables. To determine the independent risk factors for D-AKI, univariate and multivariate binary logistic regression analyses were used. The variables with significant differences in the univariate analysis were included in the multivariate logistic regression model (*P* < 0.05 was used for entry and *P* > 0.10 for removal) using the Enter mode. Estimates of odds ratios (ORs) and 95% confidence intervals (CIs) for risk factors were obtained. A value of *P* < 0.05 was considered significant. All the reported *P* values were 2-sided.

## 3. Results

### 3.1. Clinical Characteristics of the Patients

**Figure 1:**
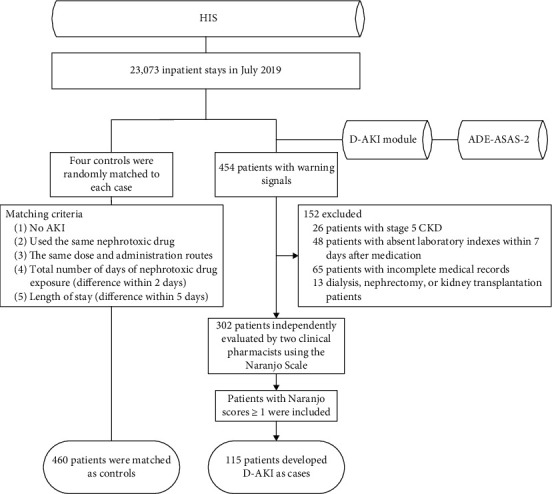
Flow chart of study population selection. HIS: hospital information system; D-AKI: drug-induced acute kidney injury; ADE-ASAS-2: Adverse Drug Events Active Surveillance and Assessment System-2; AKI: acute kidney injury; CKD: chronic kidney disease.

A total of 23,073 patients admitted in July 2019 were monitored by the ADE-ASAS-2; 1,942 (8.42%) were automatically excluded by the ADE-ASAS-2, and 454 (1.97%) elicited alarm signals. Among these patients, after independent revaluation by two clinical pharmacists using the Naranjo Scale, we finally identified 115 (25.33%) patients who were diagnosed with D-AKI, and 460 controls were matched to these patients. The cases and controls involved 26 and 32 clinical departments, respectively, of which 54/115 (46.96%) and 213/460 (46.30%) were surgical, 42/115 (36.52%) and 186/460 (40.43%) were internal medicine, 3/115 (2.61%) and 9/460 (1.96%) were emergency, and 15/115 (13.04%) and 46/460 (10.00%) were intensive care units. A total of 46 drugs were administered in the 115 D-AKI cases, and the distribution of primary suspected drugs is shown in Table 1. The top 5 drugs in terms of incidence were teicoplanin (7/132, 5.30%), meropenem (6/375, 1.60%), vancomycin (4/431, 0.93%), cefoperazone sodium and sulbactam sodium (7/832, 0.84%), and cefmetazole sodium (4/543, 0.74%). The selection process is summarized in Figure 1. The characteristics of the cases and controls are described in Table 2.

### 3.2. Risk Factors for Drug-Induced Acute Kidney Injury

The potential risk factors for D-AKI were analyzed by univariate regression and multivariate regression and are shown in Table 3. In the univariate analysis, the significant correlates included age, age ≥ 60, smoking history, alcohol abuse, number of concomitant drugs, number of concomitant drugs ≥ 15, ARBs, NSAIDS, diuretics, hypertension, diabetes mellitus, cardiovascular disease, anemia, CKD, baseline SCr, baseline eGFR, baseline eGFR < 60, fasting blood glucose, HB, RBC count, WBC count, neutrophil count, monocyte count, RDW, PLT count, and NLR. All potential risk factors (*P* < 0.05 in the univariable analysis) were evaluated in the multivariable regression analysis. In the final regression model, the following variables were included: alcohol abuse, use of NSAIDs, use of diuretics, anemia, and CKD. The independent risk factors for D-AKI included alcohol abuse (OR = 2.05, 95% CI: 1.04-4.07, *P* = 0.039), use of NSAIDs (OR = 2.39, 95% CI: 1.25-4.58, *P* = 0.009), use of diuretics (OR = 2.64, 95% CI: 1.42-4.92, *P* = 0.002), anemia (OR = 4.10, 95% CI: 1.94-8.67, *P* < 0.001), and CKD (OR = 2.33, 95% CI: 1.07-5.08, *P* = 0.033).

## 4. Discussion

In this case-control study, we identified the clinical features of patients hospitalized for D-AKI and its associated risk factors. With the help of the ADE-ASAS-2, alarm signals were triggered in 454 cases, and 302 patients were identified as having hospital-acquired AKI; among them, 115 patients (38.08%) were diagnosed with D-AKI, consistent with the results of previous studies [24, 25]. To include more cases and improve the sensitivity, a lenient exclusion trigger was set; the positive alarm rate was triggered in 25.33% of cases, better reflecting the real-world characteristics of D-AKI. This study used the ADE-ASAS-2 to simultaneously monitor all the medications of inpatients in our hospital. D-AKI was found to be associated with 46 drugs, and the incidence was calculated, significantly improving the efficacy in determining the ADR occurrence in a large sample population in a multidrug environment. Currently, AKI is still a common complication that is associated with a high mortality rate and prolonged hospital stay [26, 27]. Accordingly, studying D-AKI to determine specific risk factors is of major importance. Most existing studies have focused on general risk factors in specific patients or specific drugs [12, 28, 29] but lack a comprehensive evaluation of drug-related AKI risk factors. The main strength of this study is the exhaustive analysis of D-AKI episodes in hospitalized patients through the independently developed ADE-ASAS-2.

In this study, patients in the case group were older than those in the control group (median 64 vs. 58 years; *P* = 0.001), and a significant difference between the two groups was found in the number of patients over 60 years old. Although no significant correlation with age was found in the multivariate analysis, it has been reported as an independent risk factor for AKI in previous studies [10, 11, 28, 30, 31]. Renal structure, function, and hemodynamics change in elderly individuals, and multiple diseases often occur simultaneously, further increasing the risk of exposure to nephrotoxic drugs. Age is therefore a risk factor for D-AKI [32]. In terms of gender distribution, there was no significant difference between the case group and the control group, consistent with previous studies [29, 32–34]. In addition, we also analyzed the length of hospital stay and the number of concomitant drugs used in both groups. Previous studies have shown that for every additional nephrotoxic drug given to a patient in the same population, the risk of AKI increases by 53% [33, 35]. In the univariate analysis in this study, more than 15 concomitant drugs increased the risk of D-AKI (*P* < 0.001). In addition, a lower baseline eGFR and higher baseline SCr tended to increase the risk of renal injury [10, 11, 27, 29, 34–37]. However, we did not find that the baseline eGFR and baseline SCr were independent risk factors for D-AKI, so large-sample studies should be performed to clarify the relationship between these factors.

We observed an association between D-AKI and alcohol abuse, NSAIDs, diuretics, CKD, anemia, and neutrophil count. In our study, we found that alcohol abuse (adjusted OR = 2.05, 95% CI: 1.04-4.07, *P* = 0.039) was an independent risk factor for D-AKI. One possible mechanism is that oxidative stress leads to an excessive number of free radicals, which in turn trigger kidney tissue injury and increase inflammation [37]. Previous studies have shown that compared with no drinking, regular and occasional binge drinking were associated with 2.2-fold and a 2.0-fold higher risks of CKD progression, respectively. This association was particularly evident in patients who had decreased kidney function and proteinuria [38, 39]. In addition, we also found a significant correlation between previous CKD and D-AKI (adjusted OR = 2.33, 95% CI: 1.07-5.08, *P* = 0.033). This result is consistent with the findings of several previous studies [26, 29, 35, 36]. Regarding drug-related nephrotoxicity, including AKI and CKD [1], recent epidemiologic and mechanistic studies have suggested that the two syndromes are closely interconnected; CKD is a risk factor for AKI, AKI is a risk factor for the development of CKD [40], and both are risk factors for cardiovascular disease [10, 31]. In addition, we found that prior anemia increased the risk of D-AKI progression by 4.10-fold (95% CI: 1.94-8.67; *P* < 0.001). Anemia is associated with AKI and increased long-term mortality in critically ill patients, but the underlying mechanism is unclear; it is possible that anemia directly reduces oxygen delivery and causes kidney damage due to ischemia and hypoxia [41, 42], so kidney function in hospitalized patients with a history of alcohol abuse, CKD, or anemia should be closely monitored.

Risk factors considered specific for D-AKI include drug combinations, such as the “triple whammy,” which includes NSAIDs, angiotensin-converting enzymes, and diuretics [43–45]. In our study, the use of NSAIDs and diuretics increased the risk of D-AKI by 2.39-fold (95% CI: 1.25-4.58; *P* = 0.009) and 2.64-fold (95% CI: 1.42-4.92; *P* = 0.002), respectively. NSAIDs have been mentioned in many studies as independent risk factors for AKI [5, 26, 35]. The pathological mechanism of NSAIDs causing precipitate hemodynamically mediated kidney injury is that NSAIDs inhibit renal prostaglandins so that renal vasoconstriction preferentially occurs in afferent arterioles [31]. Diuretics, as important components of the “triple whammy,” have been shown to be nephrotoxic in many studies [5, 11, 35]. The use of high-dose diuretics could cause agitation of the sympathetic nervous system and renin-angiotensin system (RAS), leading to an increase in peripheral vascular resistance, a decrease in the left ventricular ejection fraction (LVEF), and eventually renal perfusion, resulting in AKI [10]. Therefore, serum creatinine should be monitored in inpatients administered the above drug combination to avoid the occurrence of D-AKI.

Several limitations of the study should be highlighted. First, this was a retrospective analysis with a limited level of evidence. Second, the monitoring period was only 1 month because the average number of inpatients in this hospital was more than 20,000 per month, which was enough to support this study. Follow-up studies will extend the monitoring period and minimize confounding bias associated with the admission time. Moreover, AKI was diagnosed based on dynamic changes in SCr independent of urine output, and there were cases with incomplete clinical information, which most likely resulted in missing some D-AKI cases. In addition, the risk factors for D-AKI obtained in this study overlap with the overall risk factors for AKI, and other nondrug causes may exist in D-AKI cases. Finally, several novel renal biomarkers, including neutrophil gelatinase-associated lipocalin, cystatin C, urinary Kim-1, and interleukin 18, were not measured [46, 47]. However, the sample size in this study was insufficient to adequately analyze additional variables. Therefore, more comprehensive studies with larger sample sizes are urgently needed to confirm our findings.

## 5. Conclusions

In this study, we identified alcohol abuse, the concurrent use of NSAIDs or diuretics, and previous anemia or CKD as risk factors for the development of D-AKI in hospitalized patients. Although the results require confirmation in future studies, it may be advisable to consider early initiation of prophylactic measures to prevent D-AKI in patients with these risk factors.

## Figures and Tables

**Table 1:** The distribution of the primary suspected drugs in the case group.

Drug category	*N*	Drug (*N*)
Antibiotics	50	Teicoplanin (7), cefoperazone sodium and sulbactam sodium (7), meropenem (6), ceftriaxone sodium (6), cefuroxime sodium (5), cefmetazole sodium (4), vancomycin (4), piperacillin sodium tazobactam (3), etimicin sulfate sodium chloride (2), biapenem (2), ceftazidime (2), tigecycline (1), imipenem and cilastatin (1)
NSAIDs	20	Flurbiprofen (11), parecoxib sodium (4), aspirin (3), celecoxib (1), etoricoxib (1)
Diuretics	12	Spironolactone (6), torasemide (5), furosemide (1)
Antifungal drugs	10	Caspofungin acetate (3), voriconazole (3), fluconazole (2), amphotericin B (1), amphotericin B liposome (1)
Synthetic antibiotics	8	Levofloxacin sodium chloride (3), ornidazole sodium chloride (1), compound sulfamethoxazole (1), linezolid (1), levofloxacin lactate sodium chloride (1), levofloxacin (1)
Antiviral drugs	2	Ganciclovir (1), acyclovir (1)
ACEIs	2	Fosinopril sodium (1), perindopril *tert*-butylamine (1)
Others	11	Alprostadil (2), febuxostat (1), mannitol (1), rabeprazole sodium (1), cinepazide maleate (1), loperamide hydrochloride (1), ulinastatin (1), thymus (1), paclitaxel (1), levocarnitine (1)

ACEIs: angiotensin-converting enzyme inhibitors; NSAIDs: nonsteroidal anti-inflammatory drugs.

**Table 2:** Characteristics of the cases and controls.

Variables	Cases (*N* = 115)	Controls (*N* = 460)	*P* value
*Demographic data*			
Median age (years) (range)	64 (52-73)	58 (45-68)	<0.001
Age ≥ 60, *n* (%)	72 (62.6)	216 (47.0)	0.003
Males, *n* (%)	74 (64.3)	278 (60.4)	0.441
Median height (m) (range)	1.67 (1.60-1.73)	1.69 (1.60-1.73)	0.429
Median weight (kg) (range)	63 (56-73)	65 (57-74)	0.252
BMI (mean ± SD)	23.10 ± 4.17	23.52 ± 3.70	0.280
Smoking history, *n* (%)	48 (41.7)	140 (30.4)	0.021
Alcohol abuse, *n* (%)	46 (40.0)	120 (26.1)	0.003
Median hospital stay (days) (range)	19 (11-29)	20 (10-31)	0.635
Hospital stay ≥ 15, *n* (%)	78 (67.8)	292 (63.5)	0.384
Median number of concomitant drugs, *n* (range)	19 (15-23)	16 (12-21)	<0.001
Number of concomitant drugs ≥ 15, *n* (%)	88 (76.5)	269 (58.5)	<0.001
*Medication*			
ACEIs, *n* (%)	6 (5.2)	35 (7.6)	0.373
ARBs, *n* (%)	29 (25.2)	62 (13.5)	0.002
NSAIDs, *n* (%)	42 (36.5)	94 (20.4)	<0.001
Diuretics, *n* (%)	53 (46.1)	60 (13.0)	<0.001
Vancomycin, *n* (%)	7 (6.1)	25 (5.4)	0.785
*Comorbidities*			
Diabetes mellitus, *n* (%)	35 (30.4)	75 (16.3)	0.001
Hypertension, *n* (%)	54 (47.0)	160 (34.8)	0.016
Cardiovascular disease, *n* (%)	44 (38.3)	60 (13.0)	<0.001
Anemia, *n* (%)	83 (72.2)	176 (38.3)	<0.001
CKD, *n* (%)	27 (23.5)	34 (7.4)	<0.001
*Laboratory measurements*			
Median baseline SCr (mmol/L) (range)	88.10 (60.80-117.10)	72.05 (58.75-85.88)	<0.001
Median fasting blood glucose (mmol/L) (range)	7.71 (5.95-10.38)	5.87 (5.04-7.94)	<0.001
Median UA (mmol/L) (range)	294.3 (197.1-381.4)	279.2 (205.6-346.4)	0.254
HB (g/L) (mean ± SD)	103.76 ± 26.22	113.08 ± 22.61	<0.001
RBC count (×10^12^/L) (mean ± SD)	3.43 ± 0.81	3.75 ± 0.71	<0.001
Median WBC count (×10^9^/L) (range)	9.48 (6.65-14.17)	7.26 (4.95-10.36)	<0.001
Median neutrophil count (×10^9^/L) (range)	7.65 (4.88-12.04)	5.19 (3.11-8.36)	<0.001
Median lymphocyte count (×10^9^/L) (range)	0.87 (0.55-1.35)	1.09 (0.68-1.65)	0.003
Median monocyte count (×10^9^/L) (range)	0.50 (0.34-0.77)	0.43 (0.31-0.59)	0.002
Median PLT count (×10^9^/L) (range)	153 (95-214)	190 (132-242)	0.001
Median RDW (%) (range)	14.1 (12.9-15.9)	13.2 (12.3-14.8)	0.001
Median baseline eGFR (mL/min/1.73 m^2^) (range)	80.03 (51.62-100.61)	94.54 (77.26-108.56)	<0.001
Baseline eGFR < 60 mL/min/1.73 m^2^, *n* (%)	40 (34.8)	47 (10.2)	<0.001
Median NLR (range)	8.77 (4.47-17.88)	4.74 (2.24-10.23)	<0.001
Median PLR (range)	165.08 (100.86-285.71)	158.89 (109.58-247.40)	0.808

BMI: body mass index; ACEIs: angiotensin-converting enzyme inhibitors; ARBs: angiotensin receptor blockers; NSAIDs: nonsteroidal anti-inflammatory drugs; CKD: chronic kidney disease; SCr: serum creatinine; UA: serum uric acid; HB: hemoglobin; RBC: red blood cell; WBC: white blood cell; RDW: red blood cell distribution width; eGFR: estimated glomerular filtration rate; NLR: neutrophil/lymphocyte ratio; PLR: platelet/lymphocyte ratio.

**Table 3:** Univariate and multivariate analyses of risk factors for D-AKI (cases *N* = 115, controls *N* = 460).

Variables	Unadjusted OR (95% CI)	Adjusted OR (95% CI)^∗^
Age	1.02 (1.01-1.04)	
Age ≥60	1.89 (1.24-2.88)	
Smoking history	1.64 (1.08-2.49)	
Alcohol abuse	1.89 (1.23-2.90)	2.05 (1.04-4.07)
Number of concomitant drugs	1.05 (1.02-1.08)	
Number of concomitant drugs ≥ 15	2.31 (1.45-3.70)	
ARBs	2.17 (1.32-3.56)	
NSAIDs	2.24 (1.44-3.49)	2.39 (1.25-4.58)
Diuretics	5.70 (3.61-8.99)	2.64 (1.42-4.92)
Hypertension	1.66 (1.10-2.51)	
Diabetes mellitus	2.25 (1.41-3.59)	
Cardiovascular disease	4.13 (2.60-6.57)	
Anemia	4.19 (2.67-6.56)	4.10 (1.94-8.67)
CKD	3.84 (2.21-6.70)	2.33 (1.07-5.08)
Baseline SCr	1.01 (1.01-1.02)	
Baseline eGFR	0.98 (0.97-0.99)	
Baseline eGFR <60	4.69 (2.88-7.64)	
Fasting blood glucose	1.11 (1.05-1.18)	
HB	0.98 (0.98-0.99)	
RBC count	0.55 (0.42-0.73)	
WBC count	1.13 (1.08-1.18)	
Neutrophil count	1.15 (1.09-1.20)	
Monocyte count	2.34 (1.37-4.01)	
RDW	1.09 (1.00-1.18)	
Platelet count	1.00 (0.99-1.00)	
NLR	1.02 (1.01-1.04)	

^∗^Adjusted for all variables in the table. ARBs: angiotensin receptor blockers; NSAIDs: nonsteroidal anti-inflammatory drugs; CKD: chronic kidney disease; SCr: serum creatinine; eGFR: estimated glomerular filtration rate; HB: hemoglobin; RDW: red blood cell distribution width; NLR: neutrophil/lymphocyte ratio.

## Data Availability

The data used to support the findings of this study are available upon reasonable request from the authors.

## References

[1] Awdishu L., Mehta R. L. (2017). The 6R’s of drug induced nephrotoxicity. *BMC Nephrology*.

[2] de Figueiredo T. P., de Souza Groia R. C., Barroso S. C. C., do Nascimento M. M. G., Reis A. M. M. (2017). Factors associated with adverse drug reactions in older inpatients in teaching hospital. *International Journal of Clinical Pharmacy*.

[3] Shirali A., Pazhayattil G. S. (2014). Drug-induced impairment of renal function. *International Journal of Nephrology and Renovascular Disease*.

[4] Hoste E. A., Bagshaw S. M., Bellomo R. (2015). Epidemiology of acute kidney injury in critically ill patients: the multinational AKI-EPI study. *Intensive Care Medicine*.

[5] Pierson-Marchandise M., Gras V., Moragny J. (2017). The drugs that mostly frequently induce acute kidney injury: a case−noncase study of a pharmacovigilance database. *British Journal of Clinical Pharmacology*.

[6] Taber S. S., Pasko D. A. (2008). The epidemiology of drug-induced disorders: the kidney. *Expert Opinion on Drug Safety*.

[7] Che M., Yan Y., Zhang Y. (2009). Analysis of drug-induced acute renal failure in Shanghai. *Zhonghua Yi Xue Za Zhi*.

[8] Xu X., Nie S., Liu Z. (2015). Epidemiology and clinical correlates of AKI in Chinese hospitalized adults. *Clinical journal of the American Society of Nephrology: CJASN*.

[9] Yang L., Xing G., Wang L. (2015). Acute kidney injury in China: a cross-sectional survey. *Lancet*.

[10] Wang C., Pei Y. Y., Ma Y. H. (2019). Risk factors for acute kidney injury in patients with acute myocardial infarction. *Chinese Medical Journal*.

[11] Caspi O., Habib M., Cohen Y. (2017). Acute kidney injury after primary angioplasty: is contrast-induced nephropathy the culprit?. *Journal of the American Heart Association*.

[12] Kim J. Y., Kim J. H., Yee J., Song S. J., Gwak H. S. (2018). Risk factors of opioid-induced adverse reactions in elderly male outpatients of Korea Veterans Hospital. *BMC Geriatrics*.

[13] Werner R., Horina Joerg H., Franz Q., Rosenkranz Alexander R., Gernot S. (2019). Contrast induced acute kidney injury and its impact on mid-term kidney function, cardiovascular events and mortality. *Scientific Reports*.

[14] Yang H. Y., Guo D. H., Jia W. P., Zhu M., Xu Y. J., Wang X. Y. (2019). Incidence, clinical features, and risk factors of fluoroquinolone-induced acute liver injury: a case-control study. *Therapeutics and Clinical Risk Management*.

[15] Chen C., Jia W. P., Guo D. H. (2020). Development of a computer-assisted adverse drug events alarm and assessment system for hospital inpatients in China. *Therapeutic Innovation & Regulatory Science*.

[16] Guo D. H., Su C., Wang X. Y. (2017). Research and practice of informationalized automatic monitoring on inpatients with drug-induced liver injury. *Evaluation and Analysis of Drug-Use in Hospitals of China*.

[17] Kellum J. A., Lameire N., Aspelin P. (2012). Kidney Disease: Improving Global Outcomes (KDIGO) Acute Kidney Injury Work Group. KDIGO clinical practice guideline for acute kidney injury (2012). *Kidney International Supplements*.

[18] Naranjo C. A., Busto U., Sellers E. M. (1981). A method for estimating the probability of adverse drug reactions. *Clinical Pharmacology and Therapeutics*.

[19] Mancia G., de Backer G., Dominiczak A. (2007). 2007 guidelines for the management of arterial hypertension. *Journal of Hypertension*.

[20] Mancia G., Fagard R., Narkiewicz K. (2013). 2013 ESH/ESC guidelines for the management of arterial hypertension: the Task Force for the Management of Arterial Hypertension of the European Society of Hypertension (ESH) and of the European Society of Cardiology (ESC). *European Heart Journal*.

[21] WHO Scientific Group on Nutritional Anaemias & World Health Organization (1986). *Nutritional anaemias : report of a WHO scientific group*.

[22] Mehran R., Nikolsky E. (2006). Contrast-induced nephropathy: definition, epidemiology, and patients at risk. *Kidney International*.

[23] Levey A. S., Stevens L. A., Schmid C. H. (2009). A new equation to estimate glomerular filtration rate. *Annals of Internal Medicine*.

[24] Dlamini T. A. L., Heering P. J., Chivese T., Rayner B. (2017). A prospective study of the demographics, management and outcome of patients with acute kidney injury in Cape Town, South Africa. *PLoS One*.

[25] Goswami S., Pahwa N., Vohra R., Raju B. M. (2018). Clinical spectrum of hospital acquired acute kidney injury: a prospective study from Central India. *Saudi Journal of Kidney Diseases and Transplantation*.

[26] Sales G. T. M., Foresto R. D. (2020). Drug-induced nephrotoxicity. *Revista da Associação Médica Brasileira*.

[27] Safadi S., Hommos M. S., Enders F. T., Lieske J. C., Kashani K. B. (2020). Risk factors for acute kidney injury in hospitalized non-critically ill patients: a population-based study. *Mayo Clinic Proceedings*.

[28] Motwani S. S., McMahon G. M., Humphreys B. D., Partridge A. H., Waikar S. S., Curhan G. C. (2018). Development and validation of a risk prediction model for acute kidney injury after the first course of cisplatin. *Journal of Clinical Oncology*.

[29] Rudnick M. R., Leonberg-Yoo A. K., Litt H. I., Cohen R. M., Hilton S., Reese P. P. (2020). The controversy of contrast-induced nephropathy with intravenous contrast: what is the risk?. *American Journal of Kidney Diseases*.

[30] Kobus G., Małyszko J., Bachórzewska-Gajewska H. (2019). Acute kidney injury in elderly patients. *Wiadomości Lekarskie*.

[31] Khan S., Loi V., Rosner M. H. (2017). Drug-induced kidney injury in the elderly. *Drugs & Aging*.

[32] Rosner M. H. (2013). Acute kidney injury in the elderly. *Clinics in Geriatric Medicine*.

[33] Varrier M., Ostermann M. (2014). Novel risk factors for acute kidney injury. *Current Opinion in Nephrology and Hypertension*.

[34] Sun X. P., Li J., Zhu W. W. (2018). Platelet to lymphocyte ratio predicts contrast-induced nephropathy in patients with ST-segment elevation myocardial infarction undergoing primary percutaneous coronary intervention. *Angiology*.

[35] Kane-Gill S. L., Goldstein S. L. (2015). Drug-induced acute kidney injury. *Critical Care Clinics*.

[36] Helgason D., Long T. E., Helgadottir S. (2018). Acute kidney injury following coronary angiography: a nationwide study of incidence, risk factors and long-term outcomes. *Journal of Nephrology*.

[37] Varga Z. V., Matyas C., Paloczi J., Pacher P. (2017). Alcohol misuse and kidney injury: epidemiological evidence and potential mechanisms. *Alcohol Research: Current Reviews*.

[38] Joo Y. S., Koh H., Nam K. H. (2020). Alcohol consumption and progression of chronic kidney disease: results from the Korean cohort study for outcome in patients with chronic kidney disease. *Mayo Clinic Proceedings*.

[39] Li D., Xu J., Liu F., Wang X., Yang H., Li X. (2019). Alcohol drinking and the risk of chronic kidney damage: a meta-analysis of 15 prospective cohort studies. *Alcoholism, Clinical and Experimental Research*.

[40] Chawla L. S., Eggers P. W., Star R. A., Kimmel P. L. (2014). Acute kidney injury and chronic kidney disease as interconnected syndromes. *The New England Journal of Medicine*.

[41] Sreenivasan J., Zhuo M., Khan M. S. (2018). Anemia (hemoglobin ≤ 13 g/dL) as a risk factor for contrast-induced acute kidney injury following coronary angiography. *The American Journal of Cardiology*.

[42] Han S. S., Baek S. H., Ahn S. Y. (2015). Anemia is a risk factor for acute kidney injury and long-term mortality in critically ill patients. *The Tohoku Journal of Experimental Medicine*.

[43] Loboz K. K., Shenfield G. M. (2005). Drug combinations and impaired renal function - the ‘triple whammy’. *British Journal of Clinical Pharmacology*.

[44] Fournier J. P., Sommet A., Durrieu G. (2014). Drug interactions between antihypertensive drugs and non-steroidal anti-inflammatory agents: a descriptive study using the French pharmacovigilance database. *Fundamental & Clinical Pharmacology*.

[45] Lapi F. L., Azoulay L., Yin H., Nessim S. J., Suissa S. (2013). Concurrent use of diuretics, angiotensin converting enzyme inhibitors, and angiotensin receptor blockers with non-steroidal anti-inflammatory drugs and risk of acute kidney injury: nested case-control study. *BMJ*.

[46] Alharazy S. M., Kong N., Saidin R. (2013). Serum neutrophil gelatinase-associated lipocalin and cystatin C are early biomarkers of contrast-induced nephropathy after coronary angiography in patients with chronic kidney disease. *Angiology*.

[47] Duan S. B., Liu G. L., Yu Z. Q., Pan P. (2013). Urinary KIM-1, IL-18 and Cys-c as early predictive biomarkers in gadolinium-based contrast-induced nephropathy in the elderly patients. *Clinical Nephrology*.

